# Long-term outcome of vertebral artery origin stenosis in patients with acute ischemic stroke

**DOI:** 10.1186/1471-2377-13-171

**Published:** 2013-11-11

**Authors:** Young Jin Kim, Joon Hwa Lee, Jin Woo Choi, Hong Gee Roh, Young Il Chun, Ji-Sung Lee, Hahn Young Kim

**Affiliations:** 1 Department of Neurology, Konkuk University Medical Center, Research Institute of Medical Science, Konkuk University School of Medicine, Seoul, Republic of Korea; 2 Department of Radiology, Konkuk University Medical Center, Research Institute of Medical Science, Konkuk University School of Medicine, Seoul, Republic of Korea; 3 Department of Neurosurgery, Konkuk University Medical Center, Research Institute of Medical Science, Konkuk University School of Medicine, Seoul, Republic of Korea; 4 Department of Biostatistics, College of Medicine, Korea University, Seoul, Republic of Korea

## Abstract

**Background:**

Vertebral artery origin (VAO) stenosis is occasionally observed in patients who have acute ischemic stroke. We investigated the long-term outcomes and clinical significance of VAO stenosis in patients with acute ischemic stroke.

**Methods:**

We performed a prospective observational study using a single stroke center registry to investigate the risk of recurrent stroke and vascular outcomes in patients with acute ischemic stroke and VAO stenosis. To relate the clinical significance of VAO stenosis to the vascular territory of the index stroke, patients were classified into an asymptomatic VAO stenosis group and a symptomatic VAO stenosis group.

**Results:**

Of the 774 patients who had acute ischemic stroke, 149 (19.3%) of them had more than 50% stenosis of the VAO. During 309 patient-years of follow-up (mean, 2.3 years), there were 7 ischemic strokes, 6 hemorrhagic strokes, and 2 unknown strokes. The annual event rates were 0.97% for posterior circulation ischemic stroke, 4.86% for all stroke, and 6.80% for the composite cardiovascular outcome. The annual event rate for ischemic stroke in the posterior circulation was significantly higher in patients who had symptomatic VAO stenosis than in patients who had asymptomatic stenosis (1.88% vs. 0%, p = 0.046). In a multivariate analysis, the hazard ratio, per one point increase of the Essen Stroke Risk Score (ESRS) for the composite cardiovascular outcome, was 1.46 (95% CI, 1.02-2.08, p = 0.036).

**Conclusions:**

Long-term outcomes of more than 50% stenosis of the VAO in patients with acute ischemic stroke were generally favorable. Additionally, ESRS was a predictor for the composite cardiovascular outcome. Asymptomatic VAO stenosis may not be a specific risk factor for recurrent ischemic stroke in the posterior circulation. However, VAO stenosis may require more clinical attention as a potential source of recurrent stroke when VAO stenosis is observed in patients who have concurrent ischemic stroke in the posterior circulation.

## Background

A recent systematic review revealed that the recurrence rates of stroke and vascular events in medical secondary stroke prevention trials have declined substantially over the last 5 decades [1]. For recurrent stroke, the annual rate fell from 8.7% in trials that began in the 1960s to 4.9% in trials that began in the 2000s [1].

Atherosclerosis of the vertebrobasilar artery is one of the major causes of ischemic stroke in the posterior circulation [2,3]. The most proximal portion of the extracranial vertebral artery is the second most common area of stenosis, next to the carotid bifurcation [4]. In clinical practice, stenosis of vertebral artery origin (VAO) is occasionally observed in patients who have acute ischemic stroke. In a hospital-based cohort that consisted of patients who had various atherosclerotic arterial diseases, the risk of ischemic stroke in the posterior circulation was higher in patients who had VAO stenosis than in those who did not have VAO stenosis (annual rate, 0.4% vs. 0.1%), even though the annual rate was very low [5]. However, VAO stenosis has not been well studied as a risk factor for stroke recurrence in patients with acute ischemic stroke, especially for posterior circulation ischemic stroke. Previous case series studies have reported controversial results. Stenosis of VAO could be an important embolic source for ischemic stroke in the posterior circulation [3,6-9] or could be a benign condition because there is a good collateral supply from the contralateral vertebral artery or branches of the external carotid artery [10,11]. A recent pooled analysis study indicated that symptomatic vertebrobasilar stenosis can be a strong predictor of stroke recurrence [12]. Our study was performed to investigate the long-term outcomes in patients who had acute ischemic stroke and VAO stenosis and to examine the clinical significance of VAO stenosis as a potential risk factor for ischemic stroke in the posterior circulation or other cardiovascular outcomes.

## Methods

### Patient inclusion and clinical assessments

The subjects used in this study were a subset of patients enrolled in the Konkuk Stroke Registry, a single-center prospective observational cohort study that has been described elsewhere [13,14]. We evaluated the risk of recurrent stroke and vascular outcomes in patients with acute ischemic stroke and VAO stenosis who were recruited consecutively from December 2007 to December 2010. Acute ischemic stroke was confirmed by diffusion-weighted MRI (DWI), which was performed within 5 days from the onset of stroke in patients who had been admitted consecutively to our center within 72 h after their stroke-onset. Based on the contrast-enhanced MR angiography (CE-MRA) data, trained neurologists (YJ Kim or JH Lee) determined the degree of VAO stenosis through a modified method according to the North American Symptomatic Carotid Endarterectomy Trial [15]. Patients who had more than 50% stenosis of the VAO on CE-MRA images were recruited for the study. In cases of discrepancy, a senior stroke neurologist (HY Kim) made the final decision. Patients who underwent angioplasty and stenting for VAO stenosis in the acute period of the index stroke were excluded from the study. These exclusions were made because the purpose of our study was to investigate the long-term outcomes under optimal medical treatment.

Vascular risk factors, which included hypertension, diabetes mellitus, hyperlipidemia, cardiac arrhythmia, smoking, peripheral arterial disease, and previous stroke history, were evaluated. To determine the role of multiple simultaneous risk factors, we evaluated risk profiles using the Essen Stroke Risk Score (ESRS). The ESRS was useful as a predictor of stroke recurrence; we calculated a sum score (0–9 points) based on the following risk factors: 2 points for age >75 years and 1 point each for ages 65–75 years, hypertension, diabetes mellitus, previous myocardial infarction, other cardiovascular diseases, peripheral arterial disease, current or past (<5 years) smoking, and previous transient ischemic attack (TIA) or ischemic stroke history in addition to the qualifying event [16].

Acute stroke care and optimal medical treatment for secondary stroke prevention were performed based on stroke guidelines [17]. Acute stroke care was performed in the stroke unit, and optimal medical treatment for secondary prevention included modification of blood pressure, lipid profile and glucose intolerance, treatment using antithrombotics and statins, and encouraging patients for better compliance. The primary outcome variable was recurrent stroke of any type. Secondary outcomes included composite cardiovascular events (nonfatal stroke, hospitalization for unstable angina, nonfatal myocardial infarction, and vascular death) and non-vascular death. Recurrent stroke was defined as an acute neurological event that has focal symptoms and signs consistent with a brain lesion and lasting for more than 24 h. Whenever available, a brain CT or MRI was obtained at the time of the recurrent neurological events to confirm the type of recurrent stroke. Posterior circulation ischemic stroke was defined as clinical symptoms correlated with new lesions in the brainstem, cerebellum, or occipital lobe in the vascular territory of vertebrobasilar circulation. Anterior circulation ischemic stroke was defined as clinical symptoms correlated with new lesions in the vascular territory of the anterior cerebral artery, middle cerebral artery, or internal carotid artery. Hemorrhagic stroke was defined as either a parenchymal, subarachnoid, or intraventricular hemorrhage detected on a brain CT or MRI that is associated with the recurrent neurological events. In cases where brain imaging could not be obtained by investigators, if recurrent stroke was confirmed by telephone interview, it was classified as unknown stroke. Deaths directly related to the stroke, myocardial infarction, or other vascular causes within 28 days of the patient first being seen were classified as vascular death. Patient follow-ups were conducted either by a face-to-face interview at an outpatient clinic with a physician or by a telephone interview with a trained nurse at 3 months, 1 year, and every year thereafter. Based on the mean follow-up period used in previous studies of patients who had angioplasty and stenting of the vertebral artery [18], the duration of follow-up was designed to be at least 2 years. This study was approved by the Konkuk University Medical Center Institutional Review Board as a prospective observational study.

### MR imaging study

All MR examinations were performed using a 3 T MR scanner (Signa HDx; GE Healthcare, Milwaukee, WI, USA) with an 8-channel head coil. T1-, T2-weighted, fluid attenuation inversion recovery, T2* gradient-echo, DWI, and intra- and extracranial MRA were obtained. T1-weighted, T2-weighted, and DWI were obtained during the same imaging session and in the same orientation and slice position: matrix = 512 × 224 in T1-weighted, 384 × 384 in T2-weighted and 128 × 128 in DWI; field of view = 220 × 220 mm in T1 and T2-weighted and 240 × 240 mm in DWI; section thickness = 5 mm in T1 and T2-weighted and 3.5 mm in DWI; intersection space = 2 mm in T1 and T2-weighted and 0 mm in DWI; and diffusion gradient strength, two b values = 0 and 1000 s/mm^2^. The MRA consisted of three-dimensional time-of-flight (TOF) sequences with the following parameters: repetition time (TR) = 24 ms; echo time (TE) = 2.6 ms; acquisition = 1; flip angle = 20°; matrix of 512 × 512 using zero-fill interpolation = 384 × 224; effective section thickness = 0.6 mm; and field of view = 220 × 220 mm. Additionally, CE-MRA was performed for the evaluation of neck vessels using the time-resolved method. A typical injection protocol consisted of a bolus injection of 10 mL of gadobutrol (Gadovist® 1.0; Bayer Schering Pharma AG, Berlin, Germany) at a rate of 2.0 mL/s, followed by flushing with 35 mL of saline bolus at the same rate. CE-MRA was acquired with a three-dimensional gradient echo sequence using the time-resolved method (Time-Resolved Imaging of Contrast Kinetics, TRICKS). The acquisition parameters for CE-MRA were as follows: TR/TE = 3/1.1 ms; flip angle = 20°; matrix = 320 × 192; slice thickness = 2.8 mm; field of view = 320 × 160 mm; and a bandwidth = 83.33 kHz. Three-dimensional volume acquisitions were coronally oriented with section coverage from the aortic arch to the circle of Willis and laterally to both subclavian arteries. Moreover, volume acquisitions were conducted in 13 temporal phases with a temporal resolution time of 2.4 s and a total scan time of approximately 41 s. Each three-dimensional image set was viewed as a coronal maximum-intensity projection.

### Other clinical considerations

**Figure 1:**
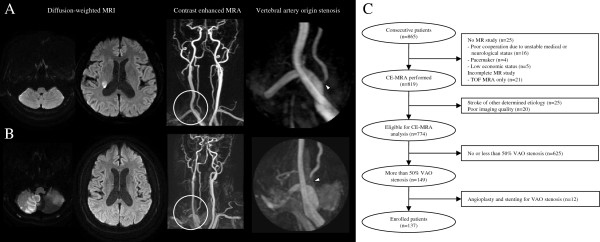
**Classification of vertebral artery origin stenosis.** Representative cases of asymptomatic stenosis of vertebral artery origin **(A)** when acute infarcts were observed in the anterior circulation and symptomatic stenosis of vertebral artery origin **(B)** when acute infarcts were observed only in the posterior circulation. Arrowheads indicate stenosis of vertebral artery origin. **(C)** Patient inclusion flowchart.

To relate the clinical significance of VAO stenosis to the vascular territory of the index stroke, patients were classified into an asymptomatic VAO stenosis group when acute infarcts were observed in the anterior circulation (Figure 1A) or into a symptomatic VAO stenosis group when acute infarcts were observed only in the posterior circulation (Figure 1B). Because symptoms of posterior circulation TIA can be vague, TIA patients who did not have acute ischemic lesions on DWI were excluded from our study. Infarcts in the vascular territory of the posterior inferior cerebellar artery (PICA) and VAO stenosis on the side contralateral to the infarct were also included in the symptomatic VAO stenosis group because PICA branching from basilar artery, anterior inferior cerebellar artery, or contralateral vertebral artery is not a rare variation [19]. However, we could not confirm these variations because we did not perform conventional angiography routinely. The presence of concurrent severe cerebrovascular atherosclerosis, including concurrent severe steno-occlusion of the contralateral vertebral artery, tandem stenosis of the intracranial vertebrobasilar artery or severe concurrent carotid stenosis, and stroke subtype were evaluated as possible risk factors for long-term outcome. Stroke subtypes were classified by two neurologists (JH Lee and HY Kim) according to the modification of the Trial of Org 10172 in Acute Stroke Treatment classification [20]. Because the aim of our study was to investigate the clinical significance of VAO stenosis, VAO stenosis was not considered as the stenosis responsible for the stroke subtype of large artery atherosclerosis. Stroke subtype in some patients who had symptomatic VAO stenosis was classified as small artery occlusion or cardioembolism instead of two or more causes if they had no other concurrent significant stenosis in the vertebrobasilar system except for VAO stenosis.

### Statistical analysis

The chi square test or Fisher’s exact test was used to compare categorical variables, and the *t* test was used to compare continuous variables, when appropriate. Data were censored at the time of outcome or at the last follow-up assessment. Annual event rates for the study outcomes were calculated as the number of outcomes divided by the patient-years of follow-up. Cardiovascular outcome-free survival was analyzed using Kaplan-Meier survival curves. Univariate and multivariate Cox proportional hazard analyses were used to identify risk factors for recurrent stroke or composite cardiovascular events. A p value of < 0.05 was considered statistically significant. Statistical analyses were performed using SPSS version 17.0.

## Results

### Patient inclusion

Of the 865 consecutive patients who had acute ischemic stroke, 25 patients (2.9%) did not undergo an MRI study because of the various following reasons: severe neurological deficits (due to unstable medical or neurological status arising from consciousness disturbance, severe dementia, or mechanical ventilator management, n = 16), use of a pacemaker (n = 4), and low economic status (n = 5) (Figure 1C). Patients who underwent TOF-MRA only, which could not cover the proximal neck vessels, were excluded (n = 21). To maintain homogeneity in patient selection, patients who had strokes of other determined etiology were excluded (n = 25). In addition, 20 patients who provided poor image quality of their proximal neck vessels were excluded from the final analysis. Among the 774 patients who were eligible for the CE-MRA analysis of VAO, 149 (19.3%) had more than 50% stenosis of VAO. Additionally, 12 patients who underwent angioplasty and stenting for VAO stenosis within 1 month after stroke-onset were excluded. The mean age was 70.4 ± 10.3 years, and other baseline characteristics are listed in Table 1. When comparing the asymptomatic and symptomatic VAO stenosis groups, concurrent vertebrobasilar stenosis was more prevalent in patients with symptomatic VAO stenosis (21.9% in the asymptomatic group vs. 38.4% in the symptomatic group, p = 0.037) (Table 1).

**Table 1:** Baseline characteristics of patients with asymptomatic or symptomatic vertebral artery origin stenosis

	**Total (n = 137)**	**Vertebral artery origin stenosis**		**P-value**
		**Asymptomatic (n = 64)**	**Symptomatic (n = 73)**	
Age, years (mean ± SD)	70.4 ± 10.3	71.5 ± 10.8	69.5 ± 9.9	0.247
Male (n,%)	73 (53.3)	40 (62.5)	33 (45.2)	0.043
Hypertension (n,%)	96 (70.1)	46 (71.9)	50 (68.5)	0.666
Diabetes (n,%)	66 (48.2)	30 (46.9)	36 (49.3)	0.776
Hyperlipidemia (n,%)	56 (40.9)	28 (43.8)	28 (38.4)	0.522
Atrial fibrillation (n,%)	6. (4.4)	4 (6.3)	2 (2.7)	0.316
Smoking (n,%)	61 (44.5)	29 (45.3)	32 (43.8)	0.862
Previous stroke history (n,%)	34 (24.8)	20 (31.3)	14 (19.2)	0.103
Coronary arterial disease	27 (19.7)	12 (18.8)	15 (20.5)	0.792
Peripheral arterial disease	5 (3.6)	2 (3.1)	3 (4.1)	0.759
ESRS (mean ± SD)	3.2 ± 1.5	3.3 ± 1.5	3.1 ± 1.4	0.339
Concurrent vertebrobasilar stenosis*	42 (30.7)	14 (21.9)	28 (38.4)	0.037
Concurrent carotid stenosis^†^	28 (20.4)	16 (25.0)	12 (16.4)	0.215
Stroke subtype				0.304
Small artery occlusion	39 (28.5)	21 (32.8)	18 (24.7)	
Large artery atherosclerosis	48 (35.0)	24 (37.5)	24 (32.9)	
Cardioembolism	11 (8.0)	6 (9.4)	5 (6.8)	
Two or more causes	16 (11.7)	4 (6.3)	12 (16.4)	
Negative evaluation	23 (16.8)	9 (14.1)	14 (19.2)	

*indicates the presence of concurrent severe stenosis or occlusion of the contralateral vertebral artery, or tandem stenosis of intracranial vertebral artery or basilar artery.

^†^indicates the concurrent presence of more than 50% stenosis of at least one carotid artery.

*ESRS* Essen Stroke Risk Score.

### CE-MRA vs. conventional angiography

Among 49 VAO stenoses (more than 50% in CE-MRA) in the 37 patients who underwent both CE-MRA and conventional angiography, 8 (16.3%) exhibited less than 50% stenosis in conventional angiography. More than 50% stenosis was concordant in the remaining 41 VAO stenoses (83.7%). However, the degree of stenosis measured by CE-MRA was somewhat overestimated compared to the degree measured using conventional angiography.

### Performance of the optimal medical treatment

As indicators of risk factor control, blood pressure, lipid profile, and HbA1c level were measured. Profiles of these indicators were improved at 3 and 12 months of follow-up. Intensity of the optimal medical treatment was also assessed by compliance to taking medication, which included antihypertensive drugs, antithrombotics, or statins. Almost all patients were on antithrombotics (> 90%), and most patients were taking antihypertensive drugs and statins (> 70%) at 3 and 12 months of follow-up. Compared to the baseline, follow-up profiles of blood pressure, total cholesterol, LDL cholesterol, and HbA1c were significantly improved in both groups (Table 2). The performance of optimal medical treatment for risk factor management and control was adequate in both groups.

**Table 2:** Risk factor control and medication assessed at 3 and 12 months of follow-up

		**Baseline**	**3-month follow-up**	**12-month follow-up**
Asymptomatic VAO stenosis (n = 64)	Systolic BP (mmHg)	151.0 ± 29.3 (n = 64)	124.5 ± 15.4 (n = 51)*	123.4 ± 13.8 (n = 42)*
	Diastolic BP (mmHg)	84.6 ± 16.0 (n = 64)	71.0 ± 10.1 (n = 51)*	71.5 ± 8.8 (n = 42)*
	Total cholesterol (mg/dl)	171.8 ± 36.7 (n = 63)		141.0 ± 34.7 (n = 49)*
	LDL cholesterol (mg/dl)	107.9 ± 29.7 (n = 59)		80.7 ± 31.3 (n = 27)*
	HDL cholesterol (mg/dl)	43.3 ± 10.5 (n = 63)		44.8 ± 11.7 (n = 48)
	HbA1c (%)^§^	8.0 ± 2.2 (n = 30)		7.0 ± 1.2 (n = 23)^‡^
	Number of evaluated patients		55	45
	Antihypertensive drug (n,%)		42 (76.4)	37 (82.2)
	Antiplatelet agent or oral anticoagulant (n,%)		53 (96.4)	41 (91.1)
	Statin (n,%)		36 (65.5)	33 (73.3)
Symptomatic VAO stenosis (n = 73)	Systolic BP (mmHg)	152.6 ± 22.6 (n = 73)	123.3 ± 16.0 (n = 57)*	125.6 ± 14.8 (n = 49)*
	Diastolic BP (mmHg)	82.4 ± 15.3 (n = 73)	69.9 ± 9.7 (n = 57)*	72.8 ± 10.6 (n = 49)*
	Total cholesterol (mg/dl)	185.8 ± 37.3 (n = 73)		148.0 ± 31.7 (n = 53)*
	LDL cholesterol (mg/dl)	112.0 ± 31.2 (n = 70)		81.0 ± 27.5 (n = 30)*
	HDL cholesterol (mg/dl)	44.9 ± 10.6 (n = 73)		47.1 ± 17.6 (n = 52)
	HbA1c (%)^§^	8.3 ± 1.7 (n = 35)		7.3 ± 0.9 (n = 22)^†^
	Number of evaluated patients		59	51
	Antihypertensive drug (n,%)		39 (66.1)	37 (72.5)
	Antiplatelet agent or oral anticoagulant (n,%)		58 (98.3)	49 (96.1)
	Statin (n,%)		45 (76.3)	39 (76.5)

Values denote mean ± SD or numbers with percentages in parentheses. *n* number of evaluated patients.

Significantly improved compared to profiles of the baseline: *p < 0.001, ^†^p < 0.01, ^‡^p < 0.05.

^§^HbA1c was measured in patients with diabetes mellitus.

*VAO* vertebral artery origin.

### Stroke recurrence and vascular outcome

**Figure 2:**
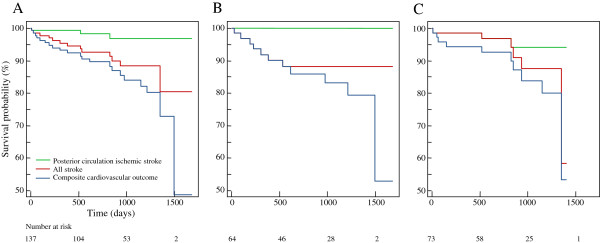
**Long-term outcome of vertebral artery origin stenosis.** The cumulative risk of posterior circulation ischemic stroke, all stroke, and the composite cardiovascular outcome of all patients **(A)**, the asymptomatic stenosis group **(B)**, and the symptomatic stenosis group **(C)** were plotted using Kaplan-Meier curves.

The mean follow-up period was 2.3 ± 1.2 years for a total of 309 patient-years. During the follow-up period, there were 7 ischemic strokes (3 in the posterior circulation and 4 in the anterior circulation), 6 hemorrhagic strokes, 2 unknown strokes, and 21 composite cardiovascular outcomes (Table 3). The annual event rates were 0.97% for posterior circulation ischemic stroke, 1.30% for anterior circulation ischemic stroke, 1.94% for hemorrhagic stroke, 4.86% for all stroke, and 6.80% for composite cardiovascular outcome (Table 3). The annual event rate of ischemic stroke in the posterior circulation was significantly higher in the symptomatic VAO stenosis group than in the asymptomatic group (1.88% vs. 0%, p = 0.046). Any other individual cardiovascular outcomes were not different between the asymptomatic and symptomatic VAO stenosis groups (Table 3). Using Kaplan-Meier curves, the cumulative risks of posterior circulation ischemic stroke, all stroke, and composite cardiovascular outcome according to the asymptomatic and symptomatic VAO stenosis groups were plotted (Figure 2).

**Table 3:** Incidence and annual event rate of outcomes during follow-up

	**Incidence, number of patients (%)**			**Annual event rate, per 100 patient-years units (95% CI)**			P-value*
	**Total (n = 137)**	**Asymptomatic (n = 64)**	**Symptomatic (n = 73)**	**Total (n = 137)**	**Asymptomatic (n = 64)**	**Symptomatic (n = 73)**	
All stroke	15 (10.9)	9 (14.1)	6 (8.2)	4.86 (2.93–8.06)	6.02 (3.13–11.58)	3.77 (1.69–8.38)	0.367
Posterior circulation ischemic stroke	3 (2.2)	0 (0)	3 (4.1)	0.97 (0.31–3.01)	0.00	1.88 (0.61–5.84)	0.046
Anterior circulation ischemic stroke	4 (2.9)	3 (4.7)	1 (1.4)	1.30 (0.49–3.45)	2.01 (0.65–6.23)	0.63 (0.09–4.45)	0.277
Hemorrhagic stroke	6 (4.4)	4 (6.3)	2 (2.7)	1.94 (0.87–4.33)	2.68 (1.00–7.13)	1.26 (0.31–5.02)	0.367
Unknown stroke	2 (1.5)	2 (3.1)	0 (0)	0.65 (0.16–2.59)	1.34 (0.33–5.35)	0.00	0.088
Unstable angina/myocardial infarction	4 (2.9)	2 (3.1)	2 (2.7)	1.30 (0.49–3.45)	1.34 (0.33–5.35)	1.26 (0.31–5.02)	0.949
Vascular death	4 (2.9)	2 (3.1)	2 (2.7)	1.30 (0.49–3.45)	1.34 (0.33–5.35)	1.26 (0.31–5.02)	0.949
Composite cardiovascular outcome	21 (15.3)	11 (17.2)	10 (13.7)	6.80 (4.43–10.43)	7.36 (4.08–13.30)	6.28 (3.38–11.66)	0.714
Non–vascular death	14 (10.2)	8 (12.5)	6 (8.2)	4.53 (2.69–7.66)	5.36 (2.68–10.71)	3.77 (1.69–8.38)	0.512

*p-value was calculated from annual event rates of the asymptomatic and symptomatic groups.

Cox proportional hazard analyses indicated that individual vascular risk factors, ESRS, symptomatic stenosis of VAO, and concurrent stenosis of other vertebrobasilar or carotid circulation were not associated with any type of stroke outcome, including posterior circulation ischemic stroke (data not shown). The presence of peripheral arterial disease and ESRS were associated with the composite cardiovascular outcome in the univariate Cox proportional hazard analyses (Table 4). Multivariate Cox proportional hazard analyses revealed that the significant association with peripheral arterial disease disappeared in model 1, and only ESRS was associated with composite cardiovascular outcome in model 2 (Table 4). The hazard ratio, per one point increase of the ESRS for the composite cardiovascular outcome, was 1.46 (95% CI, 1.02-2.08, p = 0.036).

**Table 4:** Predictors of composite cardiovascular outcome in the univariate and multivariate Cox proportional hazard analyses

	**Univariate analysis**		**Multivariate analysis**			
			**Model 1**		**Model 2**	
	**Hazard ratio (95% CI)**	**P-value**	**Hazard ratio (95% CI)**	**P-value**	**Hazard ratio (95% CI)**	**P-value**
Age (per 1 year)	1.01 (0.97–1.05)	0.720	1.00 (0.95–1.06)	0.893	-	
Sex, male	0.95 (0.40–2.23)	0.901	0.77 (0.22–2.70)	0.678	0.83 (0.34–2.05)	0.685
Hypertension	2.27 (0.66–7.75)	0.181	2.10 (0.56–7.82)	0.271	-	
Diabetes	2.21 (0.88–5.55)	0.082	1.89 (0.70–5.11)	0.208	-	
Hyperlipidemia	1.32 (0.56–3.14)	0.522	1.31 (0.52–3.33)	0.570	1.54 (0.64–3.72)	0.340
Atrial fibrillation	1.39 (0.31–6.32)	0.669	1.56 (0.24–10.24)	0.642	1.19 (0.23–6.10)	0.837
Smoking	1.19 (0.49–2.90)	0.697	1.52 (0.40–5.77)	0.538	-	
Previous stroke history	1.33 (0.51–3.44)	0.557	1.40 (0.51–3.82)	0.516	-	
Coronary arterial disease	1.26 (0.45–3.59)	0.661	0.99 (0.31–3.17)	0.984	-	
Peripheral arterial disease	4.99 (1.13–22.0)	0.034	8.29 (0.92–74.52)	0.059	-	
ESRS (per 1)	1.38 (1.01–1.88)	0.045	-		1.46 (1.02–2.08)	0.036
Symptomatic stenosis of vertebral artery origin*	0.99 (0.41–2.39)	0.978	1.09 (0.40–2.95)	0.869	1.04 (0.40–2.74)	0.935
Concurrent vertebrobasilar stenosis^‡^	1.61 (0.67–3.90)	0.287	1.12 (0.39–3.21)	0.837	1.35 (0.52–3.55)	0.540
Concurrent carotid stenosis^‡^	0.90 (0.30–2.68)	0.848	0.41 (0.89–1.91)	0.256	0.65 (0.21–2.06)	0.467

*symptomatic stenosis compared to asymptomatic stenosis of vertebral artery origin.

^†^indicates the presence of concurrent severe stenosis or occlusion of the contralateral vertebral artery, or tandem stenosis of the intracranial vertebral artery or basilar artery.

^‡^indicates the concurrent presence of more than 50% stenosis of at least one carotid artery.

*ESRS* Essen Stroke Risk Score.

## Discussion

### CE-MRA for the evaluation of VAO

Among various noninvasive techniques, recent studies have shown that CE-MRA may be the most accurate technique for evaluating stenosis of the carotid artery [21] or vertebral artery [22,23]. Compared to conventional angiography, CE-MRA may have some limitations in assessing the exact degree of stenosis at the VAO [24]. However, recent studies have shown reliable diagnostic accuracy between conventional angiography and CE-MRA, demonstrating a sensitivity of 88% and a specificity of 98% for more than 50% stenosis in the vertebrobasilar system and a sensitivity of 100% and a specificity of 85% for more than 50% stenosis at the VAO [25,26]. In our study, the concordance rate for selected patients who underwent both CE-MRA and conventional angiography was acceptable as high as 83.7%. Considering the relative invasiveness of conventional angiography, CE-MRA may be a safe and reliable alternative for the evaluation of VAO in patients with acute ischemic stroke.

### Prevalence of VAO stenosis

The prevalence of VAO stenosis is variable according to different study populations and ranges from 7.6 to 44.4% [3,5,24,27]. In a hospital-based cohort of patients with atherosclerotic arterial disease who were enrolled in the Second Manifestation of ARTerial disease (SMART) study, 282 patients (7.6%) presented asymptomatic VAO stenosis of more than 50% among a total of 3717 patients [5]. The presence of asymptomatic VAO stenosis was more frequent in the subgroup that had previous ischemic stroke history compared to the subgroup that did not (22/225, 9.8% vs. 260/3492, 7.4%) [5]. In a prospective population-based study nested within the Oxford Vascular Study, 16 patients (11.3%) had more than 50% stenosis at or near the VAO (9 at the vertebral origin and 7 near the origin) among a total of 141 patients who had posterior circulation vascular events [24]. Among 407 New England Medical Center Posterior Circulation registry patients, 131 patients (32.1%) presented more than 50% stenosis of the VAO [3]. In a non-consecutive cohort consisted of various patients who had TIA or stroke and some asymptomatic patients, the prevalence of more than 50% stenosis of the proximal vertebral artery was as high as 27.3% (33/121) in patients who had anterior circulation infarcts and 44.4% (32/72) in patients who had posterior circulation infarcts [27]. Taken together, VAO stenosis may be more prevalent in patients who have had an ischemic stroke than in patients who have had other atherosclerotic arterial diseases [5]. Among patients with ischemic stroke, VAO stenosis may be more frequently observed in patients who have had strokes in the posterior circulation compared to patients who have had strokes in the anterior circulation [27]. The prevalence of VAO stenosis (19.3%, 149/774) in our hospital-based cohort of patients with acute ischemic stroke was comparable to those reported in previous studies. In our study, the prevalence of VAO stenosis was higher in patients with posterior circulation ischemic stroke than that in patients with anterior circulation ischemic stroke (31.7%, 85/268 posterior circulation ischemic stroke vs.12.6%, 64/506 anterior or simultaneous anterior and posterior circulation ischemic stroke, p < 0.001). In particular, the prevalence of VAO stenosis in the subgroup of patients with posterior circulation ischemic stroke in our study cohort (31.7%) was comparable to those from previous cohort studies consisted of patients who had posterior circulation strokes (i.e., 11.3-44.4%) [3,24,27]. The higher incidence of VAO stenosis in patients who had ischemic strokes in the posterior circulation compared to the anterior circulation may suggest VAO stenosis as a possible cause of ischemic stroke in the posterior circulation.

### Low risk of posterior circulation ischemic stroke in patients with VAO stenosis

With the development of medical treatments, therapeutic strategies for patients who have asymptomatic carotid stenosis have recently been reevaluated [28,29]. Owing to the low recurrence rate of stroke under current optimal medical treatment, medical treatment may be more cost-effective than carotid endarterectomy or angioplasty and stenting for stroke prevention [28]. Given that VAO stenosis is a counterpart of proximal carotid stenosis in the posterior circulation, it may be interesting to compare our outcome data of the asymptomatic VAO stenosis group to data from a recent study of patients with asymptomatic carotid stenosis under the best medical treatment [29]. In this population-based study of patients who had TIA or stroke, the annual rate of ipsilateral vascular events remained quite low in 101 patients with asymptomatic carotid stenosis of more than 50% undergoing intensive medical treatment (i.e., ipsilateral TIA = 1.78% and ipsilateral stroke = 0.34%) [29]. The pattern of stroke recurrence in this asymptomatic carotid stenosis study showed trends similar to the asymptomatic VAO stenosis group of our study. The annual rate of stroke recurrence in the vascular territory of asymptomatic stenosis was very low in both studies (i.e., posterior circulation ischemic stroke in our asymptomatic VAO stenosis group = 0% vs. ipsilateral anterior circulation stroke in the asymptomatic carotid study = 0.34%). Most recurrent strokes occurred in other vascular territories unrelated to asymptomatic stenosis (i.e., all stroke in our asymptomatic VAO stenosis group = 6.02% vs. other territory strokes in the asymptomatic carotid stenosis study = 8.32%). In our study, recurrent ischemic stroke in the posterior circulation occurred only in the symptomatic VAO stenosis group, and the annual rate of posterior circulation ischemic stroke was significantly higher in the symptomatic VAO stenosis group (i.e., 0% in the asymptomatic group vs. 1.88% in the symptomatic group, p = 0.046). A higher prevalence of concurrent vertebrobasilar stenosis in the symptomatic VAO stenosis group could have contributed to the worse outcome of the posterior circulation ischemic stroke.

We compared outcomes and variates of patients enrolled in our study to a previously published study that used the same stroke registry in a similar period [14]. Patients enrolled in the previous study had acute lacunar infarcts and were comparable to acute ischemic stroke patients who did not have intra- or extra-cranial artery stenosis. The baseline characteristics of patients with acute lacunar infarcts were not different from those of VAO stenosis except for age (Table 5). ESRS was slightly higher in the VAO stenosis group, but this increase was not statistically significant. The annual event rates of outcomes were not different (Table 6). Considering the most favorable outcomes were in patients with lacunar infarcts among stroke subtypes [30], outcomes of the VAO stenosis group comparable to those of lacunar infarcts may support a hypothesis that there will be a generally favorable outcome in patients with VAO stenosis.

**Table 5:** Baseline characteristics of patients with vertebral artery origin stenosis or acute lacunar infarcts

	**Vertebral artery origin stenosis (n = 137)**	**Acute lacunar infarcts (n = 171)**	**P-value**
Age, years (mean ± SD)	70.4 ± 10.3	63.8 ± 11.5	< 0.001
Male (n,%)	73 (53.3)	97 (56.7)	0.566
Hypertension (n,%)	96 (70.1)	119 (69.6)	1.000
Diabetes (n,%)	66 (48.2)	75 (43.9)	0.491
Hyperlipidemia (n,%)	56 (40.9)	57 (33.3)	0.191
Atrial fibrillation (n,%)	6. (4.4)	5 (2.9)	0.547
Smoking (n,%)	61 (44.5)	90 (52.6)	0.170
Previous stroke history (n,%)	34 (24.8)	41 (24.0)	0.894
Coronary arterial disease	27 (19.7)	41 (24.4)	0.337
Peripheral arterial disease	5 (3.6)	6 (3.6)	1.000
ESRS (mean ± SD)	3.2 ± 1.5	2.9 ± 1.5	0.126
ESRS > 3	52 (38.0)	58 (33.9)	0.475

*ESRS* Essen Stroke Risk Score.

Published data of patients who had acute lacunar infarcts were used for the comparison; data used with personal permission [14].

**Table 6:** Annual event rate of outcomes during follow–up of patients with vertebral artery origin stenosis or acute lacunar infarcts

	**Annual event rate, per 100 patient-years units (95% CI)**		**P-value**
	**Vertebral artery origin stenosis (n = 137)**	**Acute lacunar infarcts (n = 169)**	
All stroke	4.86 (2.93–8.06)	3.38 (1.41–8.13)	0.486
Unstable angina/myocardial infarction	1.30 (0.49–3.45)	2.71 (1.02–7.21)	0.293
Vascular death	1.30 (0.49–3.45)	1.35 (0.34–5.41)	0.960
Composite cardiovascular outcome	6.80 (4.43–10.43)	6.77 (3.64–12.58)	0.990

Published data of patients who had acute lacunar infarcts were used for the comparison; data used with personal permission [14].

Considering the potential risk of procedure-related stroke or death in patients treated with angioplasty and stenting, the low absolute risk of posterior circulation ischemic stroke in patients with asymptomatic VAO stenosis who are on the best medical treatment may not warrant invasive treatment of asymptomatic VAO stenosis [5]. Because the majority of patients with asymptomatic VAO stenosis die from vascular causes other than posterior circulation ischemic stroke, therapeutic strategies should be focused on reducing the total vascular risk [5]. In a long-term follow-up study of 96 patients who had mainly VAO stenosis, recurrence of posterior circulation ischemic stroke itself was low (i.e., 2% during an average follow-up of 4.6 years) [10]. Instead, the risk of cardiovascular complications was higher in these patients than in the matched normal population [10]. In our study, individual vascular risk factors, symptomatic stenosis of VAO, or concurrent stenosis of other vertebrobasilar or carotid circulation were not associated with individual outcomes, including any type of stroke. Instead, ESRS, the sum of underlying multiple vascular risk factors, was associated with composite cardiovascular outcome.

Taken together, these results indicate that VAO stenosis itself may not be a specific risk factor for posterior circulation ischemic stroke, especially when it is asymptomatic. However, patients who have higher ESRS need more clinical attention because of the potential of more frequent future cardiovascular events.

### Angioplasty and stenting for VAO stenosis

A recent systematic review reported favorable outcomes of angioplasty and stenting in patients who had mostly symptomatic extracranial vertebral artery stenosis: i.e., posterior circulation ischemic stroke (1.3%) and TIA (6.5%) during the average follow-up period of 21 months [18]. Despite the limitation of direct comparison to the results of the systematic review, an annual event rate of 0.97% for posterior circulation ischemic stroke in our patients who had optimal medical treatment (i.e., 0% in the asymptomatic group and 1.88% in the symptomatic group) does not seem to be inferior to the historical outcome data of patients who underwent angioplasty and stenting. Although angioplasty and stenting for VAO stenosis are technically safe and feasible, data on the natural history of patients who have extracranial vertebral artery stenosis are still limited. Moreover, little is known about the most cost-effective treatment option. A randomized trial that compares medical treatment to medical treatment plus additional endovascular treatment is necessary in the future. Because individual VAO stenosis may have different clinical significance according to whether the lesion is symptomatic or not, future study subjects should be clearly defined regarding their symptomatic status for VAO stenosis.

Despite the controversial results of previous studies that focused on VAO stenosis as a possible embolic source for ischemic stroke in the posterior circulation [8,31], some patients with VAO stenosis may be at a truly higher risk for posterior circulation stroke [9,12]. Although there has been no clear consensus on the management of VAO stenosis, the presence of concurrent severe cerebrovascular atherosclerosis has been considered a reasonable indication for angioplasty and stenting under the potential high risk of recurrent ischemic stroke in the posterior circulation [8,18,32]. In our study cohort, the 12 patients who underwent angioplasty and stenting within 1 month after stroke-onset were excluded from our study. A decision for the angioplasty and stenting was based on the individual case-by-case situations: as procedures for acute intra-arterial thrombolysis in 3 patients and concurrent vertebrobasilar and/or carotid atherosclerosis in 9 patients. All of those patients had acute ischemic stroke in the posterior circulation, and they were potentially at high risk for recurrent stroke. Exclusion of those patients from the symptomatic VAO stenosis group might have biased our results. Therefore, type II error may have occurred in our study because of the small number of subjects, very low recurrence rate of posterior circulation ischemic stroke, and the exclusion of some high risk patients. However, the presence of concurrent vertebrobasilar or carotid atherosclerosis was not associated with the recurrence of posterior circulation ischemic stroke. One-sided preferences for angioplasty and stenting in patients who have VAO stenosis, simply because they have concurrent vertebrobasilar or carotid arterial stenosis, may need more careful consideration.

### Limitations of our study

The number of enrolled patients was relatively small in our study. Although the enrolled patients were prospectively collected from the stroke registry with predefined outcome follow-ups, the data were analyzed retrospectively. Therefore, some degree of bias is inevitable.

Recently, clinical concepts of optimal medical treatment have been introduced: so-called best medical treatment [29], intensive contemporary medical therapy [33], medical intervention [28], aggressive medical management [34], and others. However, specifically detailed guidelines for optimal medical treatment in a real clinical practice have not yet been established. Although we tried to provide optimal medical treatment for all patients according to the guidelines of secondary stroke prevention, our medical treatment may have not been optimal enough in some cases.

## Conclusions

Long-term outcome of more than 50% stenosis of VAO in patients who had acute ischemic stroke was generally favorable with optimal medical treatment. Additionally, ESRS was a predictor for the composite cardiovascular outcome. The clinical significance of VAO stenosis may be different according to the symptomatic status of VAO stenosis. Asymptomatic VAO stenosis may not be a specific risk factor for recurrent ischemic stroke in the posterior circulation. However, when VAO stenosis is observed in patients who had concurrent ischemic strokes in the posterior circulation (i.e., symptomatic VAO stenosis), more clinical attention on VAO stenosis may be needed because this condition could be a potential source of recurrent stroke.

## Competing interests

The authors declare that they have no competing interests.

## Authors’ contributions

HY Kim designed the study, interpreted the data, performed the statistical analyses, and drafted the manuscript. YJ Kim and JH Lee participated in the design of study, statistical analyses, and patient enrollment and helped to draft the manuscript. JW Choi, HG Roh, and YI Chun participated in patient enrollment. JS Lee helped to perform the statistical analyses. All authors read and approved the final manuscript.

## Pre-publication history

The pre-publication history for this paper can be accessed here:

http://www.biomedcentral.com/1471-2377/13/171/prepub
